# A space of goals: the cognitive geometry of informationally bounded agents

**DOI:** 10.1098/rsos.211800

**Published:** 2022-12-07

**Authors:** Karen Archer, Nicola Catenacci Volpi, Franziska Bröker, Daniel Polani

**Affiliations:** ^1^ Adaptive Systems Group, Department of Computer Science, University of Hertfordshire, Hatfield, UK; ^2^ Gatsby Computational Neuroscience Unit, University College London, London, UK; ^3^ Computational Neuroscience, Max Planck Institute for Biological Cybernetics, Tübingen, Germany

**Keywords:** information-regularized Markov decision process, decision sequences, constrained information processing, geometry, cognitive load

## Abstract

Traditionally, Euclidean geometry is treated by scientists as *a priori* and objective. However, when we take the position of an agent, the problem of selecting a best route should also factor in the abilities of the agent, its embodiment and particularly its cognitive effort. In this paper, we consider geometry in terms of travel between states within a world by incorporating information processing costs with the appropriate spatial distances. This induces a geometry that increasingly differs from the original geometry of the given world as information costs become increasingly important. We visualize this *‘cognitive geometry’* by projecting it onto two- and three-dimensional spaces showing distinct distortions reflecting the emergence of epistemic and information-saving strategies as well as pivot states. The analogies between traditional cost-based geometries and those induced by additional informational costs invite a generalization of the notion of geodesics as cheapest routes towards the notion of *infodesics*. In this perspective, the concept of infodesics is inspired by the property of geodesics that, travelling from a given start location to a given goal location along a geodesic, not only the goal, but all points along the way are visited at optimal cost from the start.

## 1. Introduction

Traditional reinforcement learning (RL) [1] is often centred around agents moving towards a specific goal. The problem of designing agents that can address multiple concurrent objectives at the same time has recently become of pivotal importance in RL [2,3].

While start and goal states are part of the same world, the resulting policies are often merely collected in the form of a ‘catalogue’ for the different start/goal combinations. Here, we wish to start from a fundamentally geometric position: we posit that in a world where the agent incurs movement costs for each action (negative rewards only), and trajectories implement directed shortest routes between a start and a goal point, these become a part of a geometry. Such a structure puts these different trajectories and the policies used to achieve them in relation with each other and allows us to systematically draw conclusions about their commonalities, what the space between these tasks looks like and how to switch between tasks or trajectories, and, finally, how to generalize solutions. The best-known form of such a structure is Euclidean geometry.

Classical Euclidean geometry is often considered as a ‘static’ concept. However, one can alternatively interpret it as the entirety of locations of a set under consideration, together with the straight lines defining the shortest routes of travel between these points, and the associated directions determined by such straight lines. In the field of cognitive science, this Euclidean notion of lines connecting bounding points has been used in the theory of conceptual spaces [4] to geometrically represent knowledge. It introduces a conceptual space which uses geometric and topological ideas to represent quality dimensions which enable one to compare concepts such as weight, colour, taste, etc. Natural categories are convex regions in conceptual spaces, in that if *x* and *y* are elements of a category, and if *z* is between *x* and *y*, then *z* is also likely to belong to the category. This convexity allows the interpretation of the focal points of regions as category prototypes. Analogous to straight lines in Euclidean space, the distance in between concepts is defined in terms of these prototypes through conceptual similarity [5] which expresses their similarity as a linear combination of their common and distinctive features.

By contrast to such a concept of ‘in-betweenness’, in traditional geometry, one has furthermore the alternative option of navigating by choosing an initial direction for a route which then is subsequently maintained in a consistent fashion. Using a compass an agent can maintain a bearing, say, a strict northern course, by selecting the desired direction and then strictly keeping its current orientation. This can be used to reach a goal, or even multiple goals, optimally whenever this fixed direction happens to coincide with one or more desired shortest routes. This concept generalizes into Riemannian geometry by replacing the straight lines with geodesics which represent a kind of consistent directionality on a manifold. This assumes that direction-constant and shortest routes coincide, otherwise they need to be treated separately.

While Riemannian geometry still respects the symmetry of distance between locations, one can generalize this to direction-dependent routes in terms of a *Finsler geometry* [6], where such distances are no longer symmetric between two given points; this models, for instance, non-reversible energy expenditure, altitude traversal or accounting for the ease of terrain. This can be elegantly expressed as RL problems which carry only positive cost (or negative reward, our convention throughout the paper). In this context, the RL policy is a decision or behaviour strategy employed by an agent to solve a task. In this paper, we will consider, among others, the limit case of an open-loop policy with optimal costs; we will argue that this can be interpreted as a generalization of selecting and following a cardinal direction by a compass while keeping the orientation fixed, which is a state-independent behaviour. More precisely, once an open-loop policy, i.e. a bearing, has been determined, using the same action or action distribution selected in every state, the agent will proceed without further deliberation or intake of information about the current state, i.e. in an open-loop manner. We interpret this as corresponding to strictly following the set direction, without adjustment.

When considering behaviour, it is traditionally hypothesized that behavioural preferences of rational agents can be characterized by a suitably chosen utility function *U* that assigns a numerical value to the possible behaviour outcomes, and the agents act as to maximize the expected value of this utility [7]. System deviations from rational behaviour are known as cognitive biases which result from the use of fast but fallible cognitive strategies known as heuristics [8]. Agents have finite cognitive resources. Thus, it is unrealistic to assume that agents would have a unique heuristic for every particular situation. Resource rationality aims to provide a framework to account for heuristics and biases, such that expected utility maximization is subject to both computational costs and cognitive limitations. In this context, acting optimally is defined as the decision strategy, or policy *π*, which maximizes the difference between the expected utility and the cost of the policy [9] according to $\pi^{*}={\arg\max}_{\pi }(E_{\pi }[U]-\mathrm{Cost}(\pi ))$. These costs include the cost of computation [10] and the cost of control [11]. The cost for the cognitive processing required to operate a given policy has also been measured in terms of information-theoretic functionals [12–15]. In the present paper, we are interested in the geometries emerging from the incorporation of such informational cognitive costs [16].

In two-dimensional (2D) continuous Euclidean spaces, the optimal routes consist of straight lines which also form shortest paths between two points. On a sphere, optimal routes lie on grand circles connecting two points; furthermore, such routes can be naturally extended beyond their boundary points, on the sphere, to full grand circles. The latter form *geodesics* which no longer necessarily constitute overall shortest routes; however, they are still *direction-preserving curves*, the generalization of the Euclidean straight line [17]. We will generally consider geometry in terms of locations (points) and optimal routes from a start location to another. Such routes are characterized by a *distance* (in our RL-based convention, negative cumulated rewards) and a *direction*.

Rethinking RL in terms of geodesics means, in some cases, splitting the resulting trajectory at a subgoal *s*′, such that the agent can visit *s*′ en route to the final goal *g* without any extra informational costs. Furthermore, instead of a cost-optimal route between two states *s* and *g*, one can instead consider the starting state *s* and a generalized ‘direction’ towards *g*, which in our case is reinterpreted as an open-loop policy.

Incorporating cognitive cost we can additionally ask how such a geometry would ideally be organized and represented in an informationally limited agent. We note that this perspective, while carrying some similarity to the questions of transfer learning [18], is still markedly distinct. No symmetry or equivariance is implied, only operational and cognitive distances (costs) and their associated policies—in particular, there is no reason to assume that one type of strategy carries over to another region. Of course, if there is some spatial coherence across regions, one can expect some relevant overlap of trajectory structures between them.

We can consider our agent starting at a given point and proceeding unchanged to ‘move in one direction’, with a fixed given behaviour, or else with a strategy optimal for a particular problem of travelling from a state *s* to *g*. Similarly to the pure cost problem, we ask which goals in addition to the original one will be optimally reached following this fixed behaviour or a least-cost route. Such generalized geometries with their directional or minimal cost routes lead us to envisage connections in the space that enable agents to adapt policies to goals beyond our current goal. Whenever the infodesic contains more than just the original start and end state, this means that multiple goals can be reached without redirection or extra costs (or with costs that do not exceed a given relaxation threshold). In the case where we consider minimal cost routes and cognitive costs incorporating the cost of processing information, we will also speak of *infodesics*. In these, constraining information processing can thus trade in nominally shorter but more complicated routes against longer, but simpler and safer ones [12,14], which effectively imposes a distortion on the geometry of a task space.

To investigate this further, we define *decision information* to quantify the amount of information processed by an agent to execute a policy to navigate from a starting location to the goal state. Here, we use an information-theoretic free energy principle [12–14,19,20], which induces a notion of informational distance and endows the decision-making problem with a qualitative geometrical interpretation.

We now introduce our formalism. After describing the simulations implemented, we present and discuss the results with suggestions for future avenues of research.

## 2. Methods

We seek to model an agent’s behaviour and the trade-off between information processing and performance. Concretely, here we model discrete gridworlds as a discrete Markov decision process (MDP). Critically, we interpret action choice in a state as the discrete analogue of geometrical ‘direction’ selection in that state.

### 2.1. Markov decision process framework

We model the system interactions between the environment and an agent ensuing from a sequence of decisions at discrete time steps for *t* ∈ 0, …, *T* using an undiscounted MDP [21] defined by the tuple <S,A,r,P>. We assume throughout that the trajectory is episodic, in particular, that the probability for trajectories generated by optimal policies to terminate at a goal state is one. At time *t*, the state of the environment is $s_{t}\in S$ (S denoting the set of states with random variable S∈S, and similarly for the set of actions A, with random variable A∈A). The policy *π*(*a*_*t*_|*s*_*t*_) denotes the conditional probability distribution $\mathrm{Pr}\{A=a_{t}|S=s_{t}\}$ which defines a stochastic choice of actions in each state *s*_*t*_ at time *t*. Note that, as appropriate, we will drop the temporal index *t* of *s* and *a* to emphasize that the policy, transition probability and reward function do not depend on time, but only on the states involved.

The dynamics of the MDP is modelled using state-action probability matrices $P_{ss^{\prime }}^{\;a}$ which define the distribution of the successor state (*s*_*t*+1_ is denoted as *s*′ where unambiguous, with random variable $S^{\prime }\in S$) given a current state and action, according to $P_{ss^{\prime }}^{\;a}\equiv p(s^{\prime }|s,a)\;:\;=\mathrm{Pr}\{S^{\prime }=s^{\prime }|S=s,A=a\}$. As a consequence of each action $a_{t}\in A$, the agent receives a reward *r*(*s*′, *s*, *a*) ≤ 0 in the subsequent time step (all the rewards from transient states are here chosen to be −1) and we refer to such an MDP as ‘cost-only’. Goal states, denoted by g∈S, are absorbing, i.e. *p*(*s*′ = *g*|*s* = *g*, · ) = 1, and *r*(*s*′ = *g*, *s* = *g*, · ) = 0; therefore, once the agent reaches a goal, no further (negative) rewards are incurred. The value function *V*^*π*^(*s*) represents the expected return of an agent when starting in a given state *s* and following policy *π* [1]. Equation (2.1) shows the value function in the form of a recursive Bellman equation which expresses the relationship between the value of a state and the values of its successor states (see [1, §3.5] and S3 in the electronic supplementary material):

2.1 $$V^{\pi }(s)=E_{\pi (a|s)p(s^{\prime }|s,a)}[r(s^{\prime },s,a)+V^{\pi }(s^{\prime })].$$

**Figure 1.**
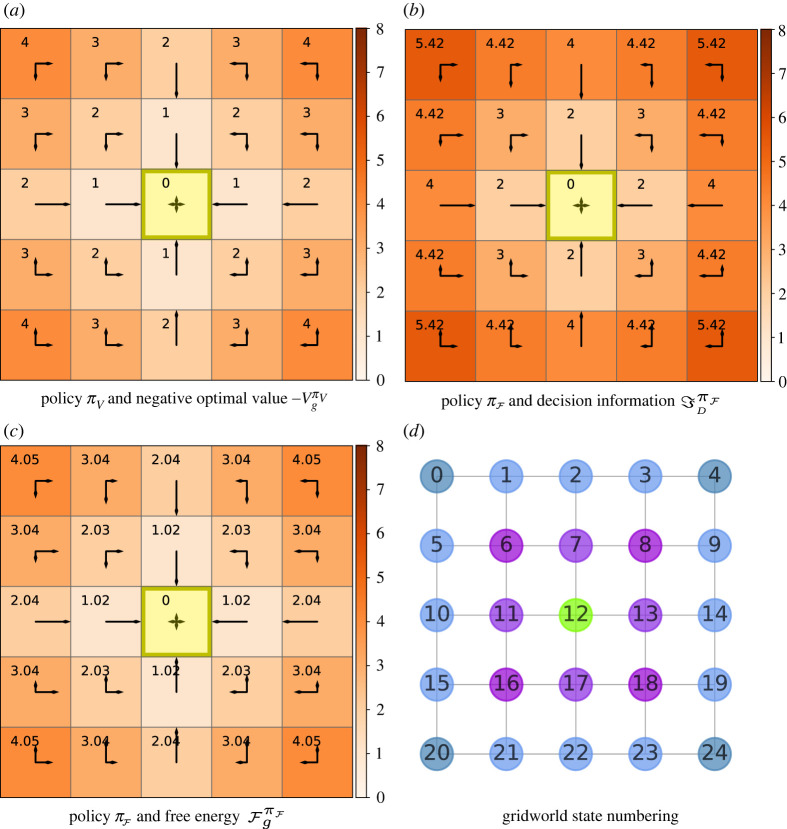
Value, decision information and free energy plots in a 5 × 5 gridworld with cardinal (Manhattan) actions A : {↑,←,↓,→}. The goal *g* = #12 is in the centre and is coloured yellow in the grid plots. The arrow lengths are proportional to the conditional probability *π*(*a*|*s*) in the indicated direction. The relevant prior, i.e. the joint state and action distribution marginalized over all transient states, $\hat{\;p}(a;\pi )$ is shown in the yellow goal state. (*a*) The policy displayed is the optimal value policy $\pi_{V}={\arg\;\max\;}_{\pi }V_{g}^{\pi }(s)$ for all s∈S. The heatmap and annotations show the negative optimal value function $-V_{g}^{\pi_{V}}(s)$ for each state. (*b*) The policy presented is optimal with respect to free energy, i.e $\pi_{F}={\arg\;\min\;}_{\pi }F_{g}^{\pi }(s;\beta )$ for all s∈S. The heatmap and annotations show decision information ${{ℑ}_{D}}^{\pi_{F}}(s)$ with *β* = 100. (*c*) The policy displayed is again $\pi_{F}$ with *β* = 100. The heatmap and annotations show free energy $F_{g}^{\pi_{F}}(s;\beta )$. (*d*) Graph showing the numbering of states in the gridworld, the goal is coloured in green and the other colours indicate levels radiating from the centre.

When following a value-optimal policy $\pi_{V}$, the agent selects a sequence of actions with the aim of maximizing the expected return, in our case minimizing the number of states visited (and actions taken) on the way to the goal. Figure 1*a* shows $\pi_{V}$ and the corresponding negative value function for a goal in the centre *g* = #12 as a heat map for a 5 × 5 gridworld with Manhattan actions. Each square in the grid is a state, with goal states denoted in yellow. We thus interpret the optimal value function $V_{g}^{\pi_{V}}$, which is traditionally written V*, as the negative distance between the agent’s position *s* and a given goal state *g*. Hence, we also denote the distance to *g* from *s* by $-V_{g}^{\pi_{V}}(s)$.

Figure 1*d* shows the gridworld represented by a graph. The nodes in this and future graphs represent states which are numbered starting from zero to |S|, and increasing first along consecutive columns and then row-wise. The colours of the nodes distinguish between different categories of states, e.g. having different locations with respect to the centre and whether or not they lie on the diagonal. The edges between the nodes indicate possible one-step transitions between states via actions. The arrows in figure 1*a*–*c* represent the policy for the agent acting in that square. The length of each arrow is proportional to the value of the conditional probability *π*(*a*|*s*), with the action *a* indicated by the arrow direction. Figure 1*b*,*c* will be explained in §§2.4 and 2.5, respectively. The numerical simulations used are detailed in §2.6.

### 2.2. The value function as a quasi-metric

We now discuss several concepts required for our geometric discussion.

A real valued function *d*(*x*, *y*) is said to be a quasi-metric for the space X if it satisfies the following conditions for all points *x*, *y*, *z* in X:

D1 : Non-negativity: *d*(*x*, *y*) ≥ 0.
D2 : Principle of indiscernibles: *d*(*x*, *y*) = 0 only if *x* = *y*.
D3 : Triangle inequality: *d*(*x*, *y*) + *d*(*y*, *z*) ≥ *d*(*x*, *z*).

We begin by showing by simple arguments that the negative optimal value function is a quasi-metric on the state space S.

As the cumulative return is non-positive *R*_*t*_ ≤ 0, for the negative optimal value we have $-V_{g}^{\pi_{V}}(s)\geq 0$, i.e. its value is non-negative for all s,g∈S, thus D1 holds.

All the rewards from transient states are here chosen to be −1, i.e. *r*(*s*′ = ·, *s* = *q*, *a* = ·) = −1 for all q∈{S∖g}, apart from the transitions where the goal is the starting state in which case the reward is zero, i.e. *r*(*s*′ = *g*, *s* = *g*, *a* = ·) = 0. This means that $-V_{g}^{\pi_{V}}(s)=0$ can only be obtained if *s* is already the goal itself, thus D2 holds.

We can express the triangle inequality for the value function as: $-V_{s^{\prime }}^{\pi_{V}}(s)-V_{g}^{\pi_{V}}(s^{\prime })\geq -V_{g}^{\pi_{V}}(s)$, for all $s,s^{\prime },g\in S$, where *s*′ is some arbitrarily chosen intermediate state. This can be easily proven by contradiction. In fact, let us assume that one had *s*, *s*′, *g* with $-V_{s^{\prime }}^{\pi_{V}}(s)-V_{g}^{\pi_{V}}(s^{\prime })<-V_{g}^{\pi_{V}}(s)$. By construction, $-V_{s^{\prime }}^{\pi_{V}}(s)$ is the shortest distance between *s* and *s*′, and $-V_{g}^{\pi_{V}}(s^{\prime })$ is the shortest distance from *s*′ to *g*. This means that one could travel from *s* to *s*′ and from *s*′ to *g* with a total cost that is less than $-V_{g}^{\pi_{V}}(s)$, but that contradicts the choice of the latter as the shortest distance between *s* and *g*.

Therefore, D3 holds.

Now, in a stochastic MDP the transition probabilities in a direction may vary from those in the reverse direction. For example, actuators may be noisier in one direction than in the other, e.g. due to friction or ratcheting effects. Therefore, the value of an arbitrary policy $V_{g}^{\pi }(s)$ is in general asymmetric with respect to *s* and *g*, which is also true for its negative. This means that, while the negative optimal value function fulfils the axioms of a quasi-metric, it will not, in general, satisfy the symmetry axiom required for it to act as a metric.

### 2.3. Information processed in a decision sequence

Up to now, we only considered the raw cost of carrying out a policy from a starting to a goal state. However, in the spirit of resource-rational decision-making, we now make the transition to information measures which consider a trade-off between performance and information, for a sequence of decisions. Such measures were introduced and used in [12,14,20,22,23]. In each case, constraints on information processing are formulated using a generalization of rate distortion (see [24, ch. 10]), permitting one to compute them via a Blahut–Arimoto-style algorithm [25,26], alternating iterations of the constrained policy and an objective function (state-action value function *Q*; see electronic supplementary material, section S3(vii)). Following [12], we compute the policy which minimizes free energy (see §2.5, equation (2.6)), for which we then compute decision information (see electronic supplementary material, algorithm 1 in S6.1). In the remainder of this section we will characterize these aforementioned measures in view of our objective to find a geometric representation. They typically differ according to the priors they use to represent any previous knowledge concerning the expectations of state and action distributions in all possible future trajectories.

*Information-to-go* [20] quantifies the information processed by the whole agent–environment system over all possible future state-action sequences. It is the KL divergence between the joint probability of future trajectories conditioned on the current state and action, and the product of the marginal state and action distributions which corresponds to the prior expectation about the future. As such, it measures the information required to enact the trajectories from a state by the whole agent–environment assuming the first action has been taken. Its contributions can be decomposed into separate components, one for decision complexity and another for the response of the environment (see electronic supplementary material, S5.2).

Subsequent to this work on information-to-go, Rubin *et al.* [14] define *InfoRL*, also referred to as ‘control information’, as the relative entropy at state *s* between the controller’s policy and a fixed prior comprised of uniformly distributed actions, i.e. ‘default plan’ (see electronic supplementary material, S5.4). InfoRL is a maximum entropy cognitive cost, and it does not take into account the reduction in cognitive cost when, for example, the goal is either East or North of every state and thus only half the action space is at all relevant in every state.

Larsson *et al.* [12] redress this limitation in their definition of *discounted information* which is aimed at optimizing the use of computational resources. A single action is chosen to maximize rewards using a fixed probability distribution over states; thus, discounted information identifies an optimal prior action distribution which minimizes the information cost across all states on average (see electronic supplementary material, S5.5). This is a discounted version of the decision complexity term of information-to-go. The prior action distribution is here calculated by marginalizing the policy over a fixed probability of states. Discounted information is the measure most similar to the measure we use below (see (2.2)), with the main difference being that it uses a fixed probability distribution over states to calculate the prior.

Mutual information regularization (MIR) [22] (see electronic supplementary material, S5.6) extends the information processed in a single decision, referred to as ‘one-step entropy regularization’ to a sequence of decisions by defining a discounted value function which incorporates the mutual information between states and actions. The optimal policy is the marginal distribution over actions under the discounted stationary distribution over states (see [23, eqns (4) and (8)]).

### 2.4. Decision information

As the remit of this work is to investigate how the space of goals transforms under information processing constraints, we would like to use an information measure which, through our choice of prior, takes into account the actual behaviour induced by the agent’s actions, or, more precisely, policy. Additionally, we wish to disregard the information cost of deviations from the marginal actions in states that will not be visited by the agent. At the same time we require a measure of the expected cognitive cost where actions in the past and future carry the same weighting, and we, therefore, obviate discounting. Information-to-go is conditioned on the first state and action pair and therefore it does not include the first decision in its cognitive cost. In addition it also includes the information processed by the environment to determine the successor state resulting from an action. Optimizing both the decision term and the environmental response term results in optimal policies which prefer short trajectories and retain a substantial element of goal-directed behaviour even when significant constraints are applied to information processing.

We thus formalize the information processed in a sequence of decisions via a quantity derived from information-to-go where we drop the conditioning on the current action and condition only on the current state. Furthermore, we extract exclusively the component which impacts the decision complexity as we are only interested in the information cost for the decision-maker. This also suppresses the goal-directedness induced by the decision complexity term. Any such goal-directedness will, therefore, be entirely captured by the value function. Formally, we define *decision information* for a policy *π* as the decision cost of an agent following *π* for future trajectories starting in *s*, as per (2.2), with more details available in electronic supplementary material, S5.3. We assume that the prior $\hat{\;p}(a;\pi )$, see (2.3), is the joint distribution of states and actions marginalized over a *‘live’ state distribution* (explained below), which is denoted by $\hat{\;p}(s;\pi )$, as shown in (2.3). We use the notation *p*( · ; *π*) to identify *π* as a parameter of the distribution.

2.2 $${{ℑ}_{D}}^{\pi }(s)\;:\;=E_{\pi (a|s)p(s^{\prime }|s,a)}\left[\log\frac{\pi (a|s)}{\hat{\;p}(a;\pi )}+{{ℑ}_{D}}^{\pi }(s^{\prime })\right]$$

and

2.3 $$\hat{\;p}(a;\pi )=\sum_{s\in S}\pi (a|s)\hat{\;p}(s;\pi ).$$

Given a policy, the ‘live’ or ‘visitation’ state distribution (see electronic supplementary material, S4) is a stationary state distribution modified for an absorbing Markov chain where the probability mass in absorbing or terminal states is suppressed to model the typical state of the system while not yet in an absorbing state (see [27, §11.2]). Without that, an unmodified stationary state distribution will result in the bulk of the probability mass being located in the goal state after a sufficiently long time, with the probability of all other states being visited tending to zero. The live distribution on the state *s* expresses the probability of finding the agent at a particular non-goal state at some random time of carrying out its policy *π*, if it originally started its trajectory uniformly somewhere in the state space, at some s∈S∖g. It measures how probable it is to find an agent at an intermediate transient state en route to the goal and thereby represents the probability of the agent having to take a decision in that particular state while its policy is ‘live’. We assume that the agent’s location follows a live state distribution to make decision information coherent with the overall *a priori* probability of the agent being in the state in question while still underway to a goal state.

The prior action distribution $\hat{\;p}(a;\pi )$ encodes all information known *a priori* about the action process where we assume that the joint action distribution can be factorized by policy-consistent distributions according to $\mathrm{Pr}(A_{t}=a_{t},\ldots ,A_{T}=a_{T})=\hat{\;p}(a_{t})\cdot \hat{\;p}(a_{t+1})\cdots \hat{\;p}(a_{T})$. We also assume that for the joint state distribution, $\mathrm{Pr}(S_{t+1}=s_{t+1},\ldots ,S_{T}=s_{T})=\hat{\;p}(s_{t+1})\cdot \hat{\;p}(s_{t+2})\cdots \hat{\;p}(s_{T})$. As in [20, §6.2], we assume that the probabilities $\hat{\;p}(s_{t+1}),\hat{\;p}(s_{t+2}),\ldots$ are time-homogeneous. Therefore, the action prior is also time-independent as it is calculated using the state prior as per (2.3). In the aforementioned literature, the prior state distribution is not parameterized by the policy *π*, but rather distributed according to a uniform distribution [12,14,20]. Using uniform distributions as the state prior has the problem that the distribution fails to take into account that some states may be unlikely to be visited, except in the case of rarely travelled trajectories, while others (e.g. close to a goal) will concentrate far more probability mass. Furthermore, in contrast to a stationary distribution, a live distribution excludes all recurrent states (here, goal states) and reflects the distribution only of states visited by the agent while the latter still needs to make decisions. It is important to note that, while at any given time step this prior does not depend on the states traversed in the previous time steps of a given trajectory, the live distribution will still reflect the overall visitation frequency of states induced by the current overall policy. Furthermore, decision information raises more costs in states in which decisions differ more from the typical behaviour(s) in the transient states visited by the policy.

Figure 1*b* shows the decision information values for each state given a policy $\pi_{F}$ which is optimal with respect to free energy which is defined below. We use the goal state specifically to represent the action marginal $\hat{\;p}(a;\pi )$ rather than the actual action selection because, regardless of actions selected in this state, the agent remains in the goal and, per definition, this does not contribute to the decision information; thus displaying any actual action selection in the goal would, therefore, not be of interest. This enables a convenient comparison with the denominator in (2.2) when interpreting decision information.

### 2.5. Constraining information processing

The traditional MDP framework assumes the agent is able to access and process all information necessary for an optimal decision. However, when information processing resources are scarce, we want the agent to prioritize the processing of essential information only. This can be achieved, for instance, by compressing state information as much as possible without compromising performance [28]. Information-theoretically, one can consider the sequence of states in the agent’s trajectory as input and the respective actions taken as output; in analogy to rate-distortion theory [24], one then seeks the lowest information rate required to reach a certain value.

Formally, we seek a solution to the following constrained optimization problem, with the desired information rate $\tilde{{ℑ}_{D}}(s)$ determining the trade-off between information processing and performance:

2.4 $$\max_{\pi (a|s)}V^{\pi }(s)\;s.t.\;{{ℑ}_{D}}^{\pi }(s)=\tilde{{ℑ}_{D}}(s).$$

For optimization, we write this as an unconstrained Lagrangian (2.5), with the Lagrangian multiplier *β* for the information rate constraint $\tilde{{ℑ}_{D}}$ and *λ* for the normalization of the policy as shown in (2.5). Here, we consider overall resource constraints for the sequence and not per-step bandwidth constraints.

2.5 $$L^{\pi }(s;\beta ,\lambda )\;:\;=\frac{1}{\beta }{{ℑ}_{D}}^{\pi }(s)-V^{\pi }(s)+\lambda \left(1-\sum_{a}\pi (a|s)\right).$$

Note that *λ* is chosen in such a way that, on optimization of the Lagrangian, the associated bracketed term disappears, which reflects the fact that the policy defines a probability distribution over the actions which is normalized. Thus, the Lagrangian reduces to the following terms, which we call the *free energy* of our problem, as seen in (2.6). Free energy is the trade-off between expected utility and decision information, i.e. the cost of the information processing (2.2) required to execute a behaviour policy *π* [12,29]. In our cost-only MDPs ${{ℑ}_{D}}^{\pi }\geq 0$ and *V*^*π*^ ≤ 0, thus their free energy is always non-negative. The trade-off parameter *β* will be strictly non-zero.

2.6 $$F^{\pi }(s;\beta )\;:\;=\frac{1}{\beta }{{ℑ}_{D}}^{\pi }(s)-V^{\pi }(s).$$

In principle one might choose a different decision information threshold $\tilde{{ℑ}_{D}}(s)$ for each state as per (2.4). However, there are both conceptual as well as practical difficulties in choosing per-state thresholds consistently [30]; we, therefore, instead follow their approach and choose a single *β* for the whole system. This corresponds to a different threshold for every state *s*, which is not only computationally convenient, but also turns out to give more immediately useful results than other schemes that were explored for per-state threshold selection (at this stage, no theoretical justification for this observation is given). With this setting, a double iteration combining the Bellman equation with the Blahut–Arimoto algorithm [26] computes the free energy (see [12] and electronic supplementary material, algorithm 1 in S6). Minimizing the Lagrangian when *β* is very small yields a policy $\pi_{F}$ where decision information becomes the dominant term in (2.5) and the agent aims to minimize information processing at the expense of increasing distance cost.

From value (2.1) and decision information (2.2), free energy inherits the form of a Bellman equation shown in (2.7) where the ‘new’ value for the single recursive step (e.g. reward addition in the case of an MDP) is combined with the sum of future rewards. Decision information and the value function are both expected values taken over all future trajectories starting from the current state *s* and following the policy *π* thereafter. Free energy, which is non-negative, is thus the expected value of a weighted combination of the information processed and rewards accumulated over all future trajectories (see (2.6)). The optimal free energy $F^{\pi_{F}}(s;\beta )$ is taken at the fixed point of this equation using the corresponding free energy optimal policy $\pi_{F}$ [12,14,20]. This policy $\pi_{F}$ is the policy which maximizes performance given a constraint on information processing. For details on finding $\pi_{F}$, see S6.1 in the electronic supplementary material. Referring again to figure 1*c* shows free energy values for a *β* value of 100, which correspond to the decision information values plotted in figure 1*b*. Here, the trade-off value is weighted strongly towards value performance; thus the behaviour strategy is close to the value-optimal behaviour shown in figure 1*a*. The free energy calculated over policy $\pi_{F}$ monotonically decreases from the current state to the goal (in expectation).

2.7 $$F^{\pi }(s;\beta )=E_{\pi (a|s)p(s^{\prime }|s,a)}[\frac{1}{\beta }\log\frac{\pi (a|s)}{\hat{\;p}(a)}-r(s^{\prime },s,a)+F^{\pi }(s^{\prime })].$$

We have disambiguated the notation regarding optimal policies, conventionally written as π*, by removing the asterisk and instead using a subscript, to distinguish $\pi_{V}$ being optimal with respect to the value function from $\pi_{F}$ being optimal with respect to free energy. Following an optimal policy from state *s* to goal state *g*, the agent is able to follow a policy, with an expected free energy at a given level of performance denoted by $F_{g}^{\pi_{F}}(s)$. For the limit case of decision information approaching zero, the policy $\pi_{F}$ converges towards the marginalized action distribution in all live states. As this is the same in all states, i.e. state independent, it is an open-loop policy. One can consider this to be the discrete-world probabilistic generalization of moving in a continuous space along a fixed direction. It additionally tries optimizing the value achievable for this open-loop policy.

### 2.6. Numerical simulations

We conducted simulations of a navigational task in a 2D gridworld modelled as a cost-only MDP with a discrete and finite state space. While here considering deterministic transitions, the framework generalizes smoothly to the non-deterministic case. Details of the algorithm and the code repository (see Data accessibility section) are included in S1 of the electronic supplementary material. Starting from an initial state, at each time step the agent selects an action representing a move in the given neighbourhood: in Manhattan, with action set A : {↑,←,↓,→}, in Moore, with action set A : {↑,↖,←,↙,↓,↘,→,↗}.

In our model, if the agent bumps into a wall, the agent does not move but still receives a reward of −1, and there is thus no additional deterrent for an agent to bump into boundaries, apart from loss of time. The edges and corners are buffering incorrect actions to some extent. Practically, this facilitates successful near open-loop policies at low *β* values where incorrect actions can be frequent.

For visualization, we used a nonlinear mapping algorithm, multidimensional scaling (MDS) [31], which, given the symmetrical pairwise distances between points in a set, projects each point onto an *N*-dimensional space such that the distances between objects are preserved as much as possible. We use the Scikit-learn MDS algorithm [32] to qualitatively assess the distance-related aspects of geometry imposed by the trade-off between value (grid distance) and information. To model cognitive distances, we compute free energies $F_{s_{j}}^{\pi_{F}}(s_{i})$ between all combinations of pairs of states, *s*_*i*_, $s_{j}\in S$, creating an *n* × *n* pairwise matrix *D*, with elements $d_{ij}\;:\;=F_{s_{j}}^{\pi_{F}}(s_{i})$. In order to visualize these distances in 2D/3D Euclidean space, *D* is symmetrized into *D*^sym^ by taking the average of the reciprocal trajectories, i.e. $d_{ij}^{\mathrm{sym}}\;:\;=(d_{ij}+d_{\;ji})/2$, hence

2.8 $$D^{\mathrm{sym}}\;:\;=\frac{\left(D+D^{T}\right)}{2}.$$

Figures S2 and S3 in the electronic supplementary material show the free energy values and optimal policies for every combination of (*s*, *g*) for *β* = 1 × 10^7^ and *β* = 0.1, respectively. Figures S4 and S5 in the electronic supplementary material show the discrepancy between the symmetrized and non-symmetrized free energy values for *β* values of 0.1 and 0.01, respectively. Asymmetry comes into play particularly in the regions between the centre and the corner states.

## 3. Results

We now elucidate the nature of decision information, proceeding to demonstrate the trade-off between performance and cognitive burden, and then present visualizations of distortions that emerge under optimal policies, showing how constraints on information processing impact the geometry of the gridworld. We later explain that we interpret structure as a collection of optimal routes or ‘desics’: either geodesics which are optimal in terms of the value function (see §4) or ‘infodesics’ which are optimal with respect to free energy and thus take information processing into account (see §5). In what follows, the optimal policy $\pi_{F}$ is that which minimizes the free energy, and $\pi_{V}$ the special case when we maximize the reward (minimize the number of steps to reach the goal), i.e. the limit case obtained by setting *β* → ∞ in the free energy.

### 3.1. Decision information

**Figure 2.**
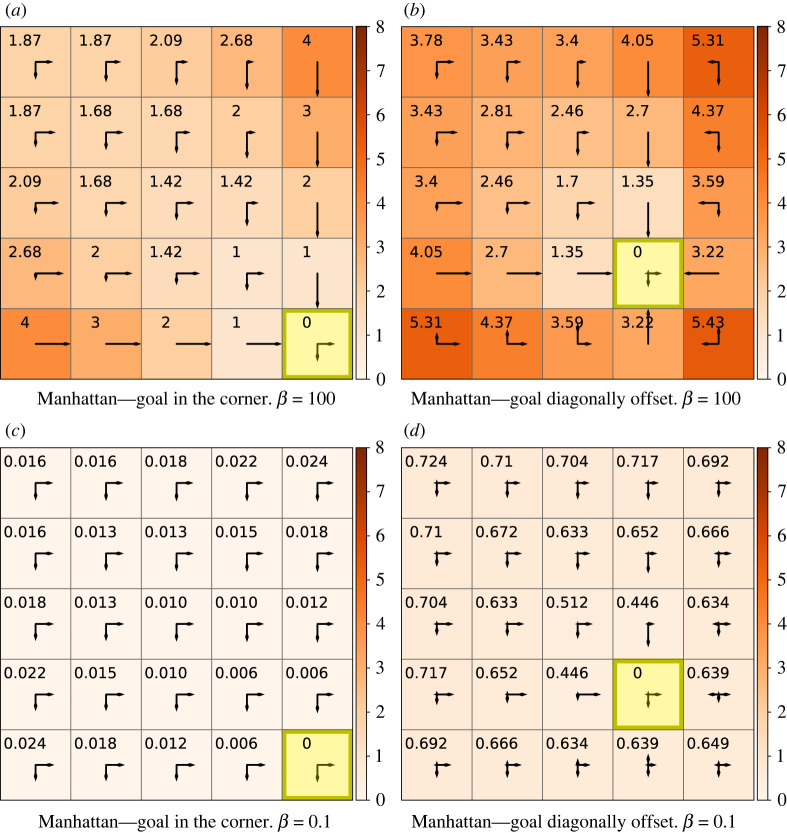
Decision information heat map in a 5 × 5 gridworld with A : {↑,←,↓,→}. Annotated values show ${ℑ}_{D}^{\pi_{F}}(s)$ in bits. The policy presented is optimal with respect to free energy, i.e $\pi_{F}={\arg\;\min\;}_{\pi }F^{\pi }(s;\beta )$. The arrow lengths are proportional to the conditional probability *π*(*a*|*s*) in the indicated direction, for convenience the prior $\hat{\;p}(a)$ is shown instead of the policy in the yellow goal state. Reward-maximizing behaviour (*β* = 100): in (*a*) the goal is in the corner and in (*b*) the goal is on the diagonal between the corner and the middle. Behaviour where information processing is constrained (*β* = 0.1): (*c*) the goal is in the corner and in (*d*) it is diagonally adjacent to the corner.

Decision information computes the information processed for the *whole* sequence of decisions leading the agent to the goal. Thus it depends on the length of the sequence. This accumulation of cost is readily seen in figure 2*a* on the trajectory moving outwards from the goal along the lower edge, where each action adds one bit to the information processing cost and longer routes thus become informationally more expensive.

From (2.2), we see that decision information increases when the alignment between the policy in a particular state and the marginalized action distribution $\hat{\;p}(a;\pi )$ decreases. Figure 2*b* shows decision information for a goal state in between the lower right corner and the middle of the grid, *g* = #18. The reader is advised to refer to figure 1*d* for state numbering. Starting from below or right of the goal, the agent is required to select actions which oppose the prevailing actions in the marginalized action distribution, causing a notable increase of decision information in these squares. For example, consider the decision information in the states adjacent to the goal: ${{ℑ}_{D}^{\pi_{F}}}_{g=\#18}(23)=3.22$ bits directly below the goal, in contrast to ${{ℑ}_{D}^{\pi_{F}}}_{g=\#18}(13)=1.35$ bits directly above the goal.

Decision information is asymmetric in general as it is a function of the policy and thus dependent on the information processing required for a particular goal, for example, moving from the corner *s* = #24 to the centre *g* = #12 carries a decision cost of ${{ℑ}_{D}^{\pi_{F}1}}_{g=\#12}(24)=5.42$ bits (figure 1*b*), in comparison to ${{ℑ}_{D}^{\pi_{F}2}}_{g=\#24}(12)=1.42$ bits from the centre *s* = #12 to the corner *g* = #24 (figure 2*a*). The cost of accurately stopping exactly in the centre is higher than stopping at the edge where some of the cost is borne by the embodiment as the agent is blocked by the wall from moving beyond the goal in the corner.

### 3.2. Visualizing cognitive geometry

**Figure 3.**
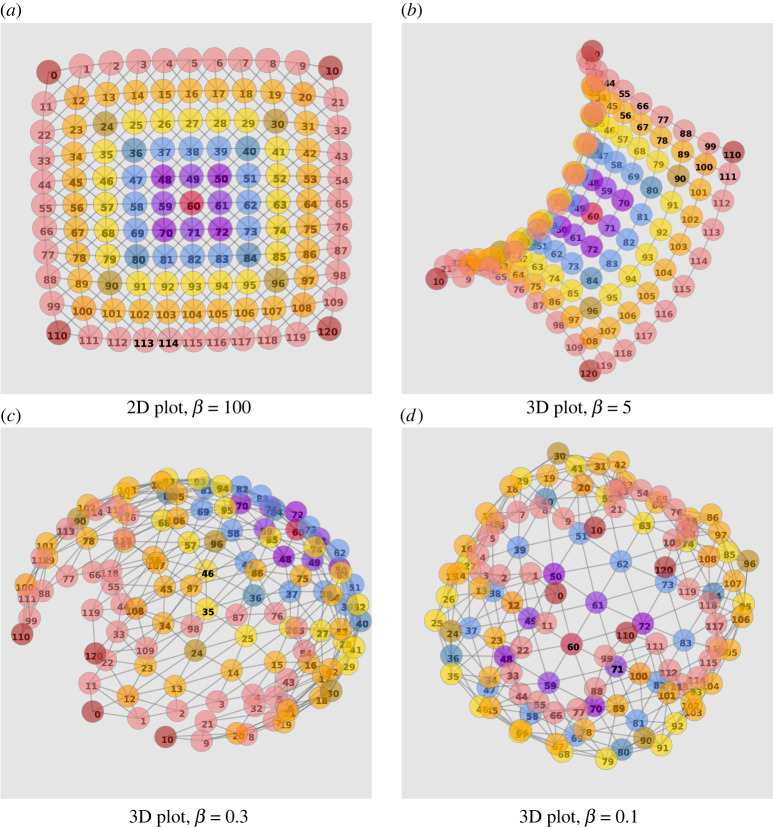
MDS visualization of the cognitive geometry induced by free energy in a Moore 11 × 11 gridworld. As *β* decreases, the corners migrate towards each other and the topology morphs from a flat grid to a mesh wrapped over a ball. (*a*) At *β* = 100, the free energies approach the optimal value function V*. With the reduction of *β*, (*b*) *β* = 5, (*c*) *β* = 0.3 and (*d*) *β* = 0.1. As a result of the constrained information processing, the policy results in similar actions taken in multiple states, i.e. the agent moves in consistent directions across the grid. The routes to the corners and along the edges are more informationally efficient, with the effect that corners migrate towards each other, despite being farthest away from each other in the original grid distance.

Cognitive geometry shapes the way agents represent the distance between states of the environment when information costs are taken into account. By way of introduction to our discussion on generalizing geodesics to rationally bounded agents (see §6.1) we begin by presenting visualizations of our cost-only gridworlds subjected to information constraints over a range of *β* values. Figure 3 shows 2D and 3D MDS embeddings for the symmetrized free energy pairwise distances (2.8) in an 11 × 11 gridworld with a Moore neighbourhood for different *β* values. In essence, free energy trades off distance travelled against the specificity of the policy as regards the state. The balance between these two factors is determined by the value of *β*. So when states are close together in terms of free energy then one needs to interpret this in light of the trade-off parameter *β*. If an agent behaves optimally with respect to value alone (approximated by *β* = 100), then the free energy reduces essentially to the distance travelled between the states, reconstituting simply the original Moore geometry, as indicated in figure 3*a*, where the underlying grid is prominently discernible. The difference between the original and the symmetrized free energies also varies with *β* as the informational component is dependent on the policy. The symmetrized free energy adjacency matrix is merely used to provide a suitable approximation for its visualization. The discrepancies between real and symmetrized free energies are shown in section S8 of the electronic supplementary material in figures S4 and S5.

In our gridworld configuration, an agent can take repeated actions to move diagonally and also get additional guidance along the edges, as bumping into them incurs only a minor time penalty. Thus the wall acts as a guide or ‘funnel’ buffering some wrong actions on the way to the corner goal. In contrast, to reach a middle goal, any wrong action is likely to move the agent away from the goal. Thus much more accurate control is required to reach precisely the middle goal compared to drifting into a corner. The latter decision strategy can be likened to *ecological rationality*, where an agent makes use of simple heuristics which specifically exploit the structure of the environment [8].

Note that, if walking into walls would be penalized, the result would be substantially different. In this case, free energy distances would increase rather than decrease closer to walls and corners would generally tend to be further away (‘pushed out’) in the free energy metric rather than closer together (‘pulled in’) as they are in our experiments. We also emphasize that the insertion of any obstacles in the world will create various, not necessarily intuitive distortions which would reflect which task groups would now become easier or more difficult to do together.

Since the informational cost of executing an optimal policy thus depends drastically on the nature of the goal, our distance, once taking this informational cost into account, will significantly distort the geometry of the gridworld as compared to a naive spatial map. When information processing is restricted via progressively lower *β* values, it becomes the cost of information processing that dominates the optimization compared to the extra time cost for bumping into the wall, and policies which use the guiding property of the edges are increasingly preferred. Thus, states on the edges, even more so in the case of the corners, effectively become closer in terms of free energy ‘distances’, as if the spatial geometry were wrapped around a ball with the corners drawn together (figure 3*c*,*d*).

## 4. Value geodesics

Armed with the observation that, given the reward function as defined previously for cost-only MDPs, the value function forms a quasi-metric over the state space (§2.2), we now define a *value geodesic* from *s* to *g* to be a sequence of arbitrary finite length *τ*_*s*→*g*_ = 〈*s*_0_ = *s*, *s*_1_, *s*_2_, …, *s*_*N*_ = *g*〉, $s_{i}\in S$ such that all elements of the sequence turn a generalization of the triangle inequality into an equality, i.e.

4.1 $$V_{g}^{\pi_{V}}(s)=\sum_{i=0}^{N-1}V_{s_{i+1}}^{\pi_{V}}(s_{i})\;\text{with}\;s_{i}\in \tau_{s\to g}.$$

Such a value geodesic does not in general consist of a contiguous sequence of states; however, in the case of cost-only MDPs, they are. Furthermore, every state *s*′ on the value geodesic lies on a shortest path from the initial state *s* to that intermediate state *s*′. So, not only is the path from *s*′ to the goal *g* optimal (this is a consequence of the Bellman property), but also the path from *s* to the intermediate states *s*′. This has an important consequence: each state on this sequence can be considered a potential alternative goal that can be reached optimally from *s* while en route to *g*. It means that benefits from reaching these intermediate goals can be ‘scooped up’ on the way to the main goal. Within this sequence, the value is strictly monotonically increasing, i.e. *V*_*g*_(*s*_*n*_) < *V*_*g*_(*s*_*m*_) for *n* < *m*.

For cost-only MDPs, the policy in the value functions of different subsegments of a value geodesic can be taken to be the same. This is because being the (negative of the) value, a quasi-metric, and given that the geodesic is the shortest path from *s* to *g* which can be traversed using the optimal policy $\pi_{V}$, then also every subsegment from *s*_*i*_ to *s*_*j*_ of this path is also the shortest path from *s*_*i*_ to *s*_*j*_ that can be traversed using the same policy $\pi_{V}$. As opposed to this, in the next section we will show for infodesics, which take into account the cost of information processing, in general each subsegment has a different optimal policy.

## 5. Infodesics

Motivated by the concept of value geodesics, we propose an analogous concept in our space where distances are determined partially or completely by an information-theoretic component. We term this concept *infodesics*. We will first consider *pure infodesics* as sequences of states that can be visited by the agent using no state information at all, i.e. with action selection being independent of state. The intention is that, as with traditional geodesics, following infodesics one obtains all interim states optimally, at no extra decision cost after choosing the initial direction (i.e. optimal open-loop policy in our perspective). In a discrete setup, we do not have intermediate directions, and since we are not using memory to enforce particular movement patterns (e.g. moving diagonally in a Manhattan world by alternating taking one step north followed by one-step east, for instance), a pure geodesic would be most closely represented by a fixed action distribution over all states. Now, decision information measures the deviation from the action marginal, i.e. from a state-independent policy; thus, its minimization corresponds to the attempt to minimize the cumulative expenditure in information processing due to deviating from that marginal. Hence, decision information can be considered a quantity that, when minimized, tries to approximate following the same direction in the continuum with as little cognitive correction as possible.

We now define a *cost infodesic* as a value geodesic where the value function *V* has been replaced by some cost function, in our case, the free energy function F (with the sign suitably reversed). In such a cost infodesic, action selection is not in general restricted to being state independent. A cost infodesic in a discrete MDP is thus an arbitrarily, but finitely long sequence *ψ*_*s*→*g*_ = 〈*s*_0_ = *s*, *s*_1_,…, *s*_*N*_ = *g*〉, $s_{i}\in S$ such that, for that sequence, all elements of the sequence turn a generalization of the triangle inequality for free energy into an equality (5.1). Formally, we demand for a cost infodesic that

5.1 $$F_{g}^{\pi_{F}}(s)=\sum_{i=0}^{N-1}F_{s_{i+1}}^{\pi_{F}^{\left(i\right)}}(s_{i})\;\text{with}\;s_{i}\in \psi_{s\to g}\;\text{by abuse of notation}.$$

The value of $F_{g}^{\pi_{F}}(s_{i})$ for such a cost infodesic is strictly monotonically decreasing as shown below:

$$F_{g}^{\pi_{F}^{\left(n\right)}}(s_{n})>F_{g}^{\pi_{F}^{\left(m\right)}}(s_{m})\;\text{for all}\;n<m.$$

Note that we do not require that the cost infodesic is contiguous (i.e. consisting only of adjacent states) throughout the trajectory, as is the case for cost-only MDPs in deterministic value geodesics; also, non-trivial infodesics (i.e. infodesics containing more than start and goal state) do not need to exist, either. They, therefore, typically constitute a strongly impoverished collection of trajectory fragments compared to traditional deterministic geodesics.

These cost infodesics, with free energy as a distance measure, constitute our objects of interest. The policy $\pi_{\;F}$ encodes a single behaviour strategy for an agent to travel from *s* → *g* resulting in a route optimal in terms of free energy. An infodesic from *s* → *g* can be split into subsegments (which we will index by *i*), which have an equivalent combined free energy cost. If these subsegments have different information costs, then the optimal policies for these subsegments are in general not equivalent and are thus distinguished using a superscript as in $\pi_{F}^{\left(i\right)}$.

When following the policy that minimizes $F_{g}^{\pi_{F}}(s)$, given the stochasticity of the policy, the agent is not guaranteed to pass through any particular intermediate infodesics states between *s* and *g* before reaching the goal. In order to ensure that the agent visits these intermediate states, subgoals need to be added to the navigation task. Optimality on the subpaths *s*_*i*_ to *s*_*i*+1_ can be achieved by allowing the agent to use multiple policies that minimize the free energies $F_{s_{i+1}}^{\pi_{F}^{\left(i\right)}}(s_{i})$. Hence, if, for instance, in the infodesics 〈*s*, *s*′, *g*〉 we make *s*′ a subgoal (i.e. the trajectory of the agent is constrained to pass though *s*′), the trajectory from *s* to *g* can be formed by concatenating the sub-trajectory from *s* to *s*′ with the sub-trajectory from *s*′ to *g*. Interestingly, when the triangle equality for free energy is thus enforced as a geodesics criterion, this concept of *policy switching* and a collection of corresponding informationally salient midpoints emerges via the segmentation of the agent’s behaviour.

We interpret geometry as essentially the whole collection of ‘desics’ under consideration, i.e. directed trajectories which connect their points in a (locally) optimal manner, according to the chosen measure. When one operates with a quasi-metric in the background, one obtains a traditional collection of geodesics, which together with their starting and goal states show which goals can optimally be scooped up en route. In our picture this collection then constitutes the geometry of the given space. Now, however, we derive the infodesics from the free energy which we later show is not a quasi-metric in general. Thus, several of the guaranteed properties of geodesics are lost. Furthermore, this collection of infodesics is much sparser, and the infodesics themselves can be ‘deficient’ compared to traditional geodesics in the sense that the infodesics are neither necessarily contiguous, nor even non-trivial. Nevertheless, the geometry still represents the collection of the various interrelations, even if now with weaker guarantees.

To regain some of the richness of structure of the traditional geodesics, we will, therefore, compensate for the above limitations by treating the free energy as a ‘deficient’ quasi-metric and permit some relaxation of constraints.

To explore the analogy with the set of traditional geodesics, we reiterate the idea: a geodesic is characterized by subsequences for which the concatenation of each section turns the triangle inequality into an equality.^1^ For the infodesics, we proceed in the same way, but since they are no longer guaranteed to be contiguous, or even non-trivial at all, in our experiments, we instead perform an exhaustive search over sequences formed by every non-repeating combination of sequences of 3, 4 or 5 states. As in the geodesic case, the states in this sequence are taken to be interim goals; free energy and respective optimal policies were calculated for each sub-trajectory within the sequence. It is important to reiterate that in the context of interim goals, the agent might follow a different optimal policy $\pi_{F}^{\left(i\right)}$ for each separate portion of a trajectory (as is also possible in the case for traditional geodesics split into segments). Since information processing cost plays a major role, this observation will turn out to be significant, not only because policies vary in terms of their informational processing costs, but because switching policies introduces additional cognitive costs which do not have an analogue in traditional geodesics.

In our setup, strict infodesics may be trivial, i.e. may end up not containing any intermediate states. We, therefore, relaxed the generalized triangle inequality requirement for the infodesic and defined an ε*-infodesic* such that the normalized difference between the sum of the free energy for the trajectory with interim goals and the total, single-goal free energy $F_{s_{T}}^{\pi_{F}}(s_{0})$ is within the range of ε, with 0<ε≪1 via (5.2). As we will show later, this normalized difference, however, can become negative, as is the case, discussed below, when, in the absence of considering the costs to switch between different policies, it becomes advantageous to use multiple policies.

5.2 $$-\varepsilon <\frac{\left[\sum_{i=0}^{T-1}F_{s_{i+1}}^{\pi_{F}^{\left(i\right)}}(s_{i})]-F_{s_{T}}^{\pi_{F}}(s_{0}\right)}{F_{s_{T}}^{\pi_{F}}(s_{0})}<\varepsilon .$$

### 5.1. Examples of ε—infodesics on cost-only Markov decision processes

**Figure 4.**
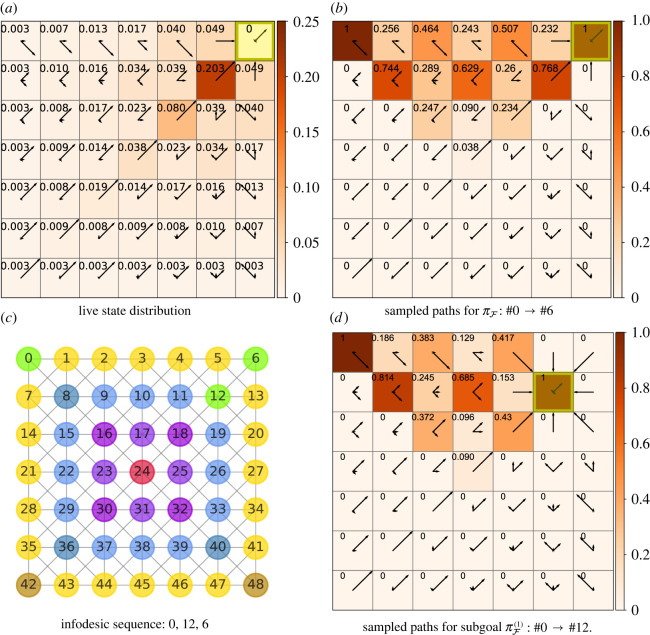
Infodesic 7 × 7 gridworld with the Moore neighbourhood and the goal in the corner state #6 and *β* = 100. (*a*) A heat map showing the live state distribution, with the policy distribution denoted by arrows of length proportional to $\pi_{F}(a|s)$ in the direction of the action. (*b*) The proportion of sampled sequences, comprised of contiguous states, for an agent following a single policy, $\pi_{F}$, from *S* = #0 which pass through various states en route to the final goal *S* = #6. (*c*) A lookup grid with states labelled with their indices and infodesic sequence states highlighted in green. The deviation from the triangle inequality is given by the normalized free energy difference which is −0.0005. We observe informationally efficient states on the diagonal; furthermore, the policy guides the agent towards these states even if it requires the agent first navigating away from the edges. (*d*) The proportion of subgoaled sampled sequences, comprised of contiguous states, for an agent following a subgoal policy, $\pi_{F}^{\left(1\right)}$, from *S* = #0 which pass through various states en route to the subgoal *S* = #12.

**Figure 5.**
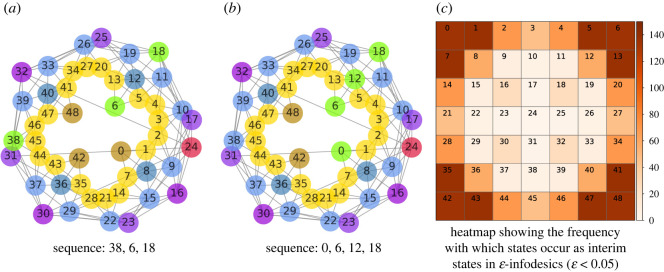
Corner states as interim states in infodesics. The environment is a 7 × 7 gridworld with a Moore neighbourhood and trade-off value *β* = 0.07. (*a*) Infodesic sequence 〈#38, #6, #18〉, with normalized free energy $(F_{18}^{\pi_{F}}(38)-[F_{6}^{\pi_{F}^{\left(1\right)}}(38)+F_{18}^{\pi_{F}^{\left(2\right)}}(6)])//F_{18}^{\pi_{F}}(38)$ =−0.187. (*b*) #0, #6, #12, #18 with free energy $(F_{18}^{\pi_{F}}(0)-[F_{6}^{\pi_{F}^{\left(1\right)}}(0)+F_{12}^{\pi_{F}^{\left(2\right)}}(6)+F_{18}^{\pi_{F}^{\left(3\right)}}(12)])/F_{18}^{\pi_{F}}(0)=-0.197$. (*c*) A heat map of the number of times a state participates as an interim state in a three-state ε-infodesic of the form $\psi_{s_{0}\to s_{g}}=\langle s_{0}=s,s_{1}=s_{i},s_{2}=s_{g}\rangle$ with ε<0.05. The cell annotations show the numbering of the states.

**Figure 6.**
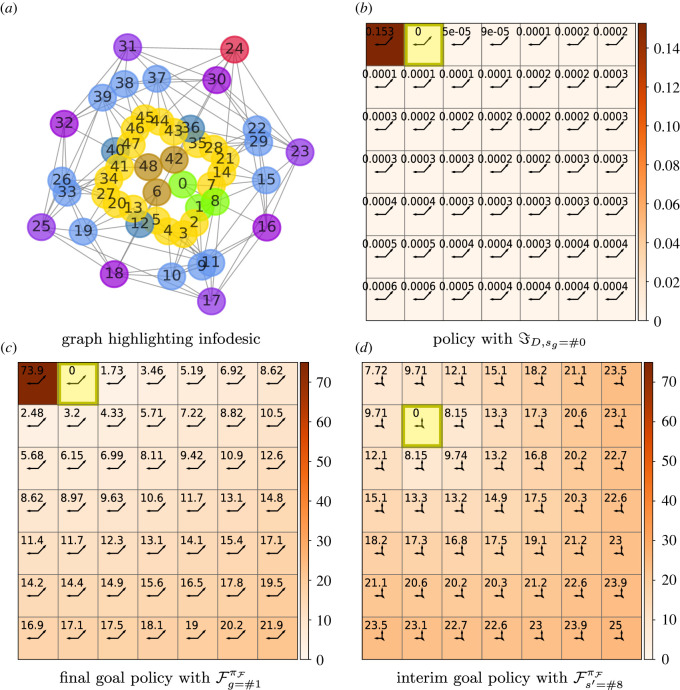
Approaching open-loop policies with *β* = 0.01. A 7 × 7 Moore gridworld with near-minimal information processing, *β* = 0.01. The agent starts in the corner #0 and aims to reach the adjacent state #1. (*a*) Graph plot showing the ε-infodesic, *ψ*_0→1_ = 〈#0, #8, #1〉, highlighted in green. (*b*) The policy and decision information for the final goal state #1, highlighted in yellow and (*c*) the same gridworld policy and goal with free energies as annotations and heatmap. (*d*) The policy and free energies for the interim goal #8, highlighted in yellow.

We study cost infodesics in a 7 × 7 Moore gridworld with three sets of examples in order of decreasing values of *β*: reward-maximizing behaviour, at *β* = 100; limited information processing, at *β* = 0.07; and near-minimal information processing, at *β* = 0.01. Figures 4–6 present these infodesics as sequences of not necessarily contiguous interim states which are highlighted in green. Free energies of trajectories, i.e. $F_{g}^{\pi_{F}}(s)$, are denoted by the starting *s* and goal *g* states. Where symbols are replaced with indices, these indices are consistent with the grid state numbering. For example, the state in the top right corner is referred to by index 6 as clarified by figure 4*c*.

The plots in figure 4 relate to a near-optimal agent (*β* = 100) navigating to a final goal in the corner (*S* = #6). Figure 4*a* shows the annotated heat map as values of the live distribution $\hat{\;p}(s;\pi_{F})$ and the corresponding optimal policy $\pi_{F}$ as arrows. In states on the diagonal between the goal and the opposite corner, the distribution of actions is similar to the marginalized action distribution (displayed in the yellow goal state) and are thus informationally more efficient. The policy of states not on this diagonal favours actions which are either parallel with the diagonal or move the agent towards this diagonal. This diagram shows that the probability mass of the state distribution is maximal at state *S* = #12 which is diagonally adjacent to the goal, with $\hat{\;p}(S=\#12;\pi_{F})≃0.2$. The next largest concentration of probability mass is $\hat{\;p}(S=\#18;\pi_{F})=0.08$, also on the diagonal. This demonstrates that the policy guides the agent towards the informationally efficient states on the diagonal en route to the goal.

An example for a cost ε-infodesic is the sequence $\psi_{s_{0}\to g}=\langle \#0,\#12,\#6\rangle$ starting from corner state *s*_0_ = #0 and ending in another corner along the same edge (*g* = #6). Its normalized free energy difference

$$\frac{\left(F_{12}^{\pi_{F}^{\left(1\right)}}(0)+F_{6}^{\pi_{F}^{\left(2\right)}}(12)\right)-F_{6}^{\pi_{F}}(0)}{F_{6}^{\pi_{F}}(0)}=-0.0005,$$

in other words, practically respecting the equality (5.1); this thus approaches an infodesic as defined in (5.1). We sampled actual paths, comprised of contiguous states, of an agent following $\pi_{F}$ en route from the left corner (*S* = #0) to the goal in the right corner (*S* = #6); the proportion of these sequences which pass through each state is shown in figure 4*b*. This confirms that, in agreement with the live distribution in figure 4*a*, the majority of paths visit the state diagonally adjacent to the goal (*S* = #12); however, as multiple ε-infodesics exist between state and goal pairs, it is not guaranteed that following a single policy will hit the intermediate state of a particular infodesic. This requires subgoaling as shown in the sampled paths of an agent following subgoal policy $\pi_{F}^{\left(1\right)}$ in figure 4*d*.

In the simple gridworlds investigated, where the boundary serves as a cost saving rail and the only consequence of hitting the boundary is a delay, as information is increasingly more constrained we have observed that corner states move closer towards each other in terms of free energy. We, therefore, look at the prevalence of corner states as interim states in cost infodesics, that is, when it is expeditious to have the agent move first to a corner before continuing to the final goal. It is important to note that this is a property of the present cost regime. If instead a substantially larger penalty were applied, then walls and corners would be avoided, especially for low values of *β* since with less information processing, the behaviour is more erratic.

Figure 5*a*,*b* shows two near-pure infodesics (with almost open-loop policies) for an agent with restricted information processing (*β* = 0.07). The goal in both cases is diagonally adjacent to the middle of the grid (*S* = #18). For the infodesic displayed in figure 5*a*, a starting state was arbitrarily chosen on the opposite side of the grid (*s*_0_ = #38). For the second infodesic, figure 5*b*, the starting state is a nearby corner state (*s*_0_ = #0). In each case it is informationally cheaper to have the agent first move into the corner adjacent to the goal (*s*′ = 6). Figure 5*b* extends the infodesic to travel outwards along the central diagonal to the final goal, $\psi_{s_{0}\to g}=\langle \#0,\#6,\#12,\#18\rangle$.

In general the agent uses the corners as cheaper waypoints. In figure 5*c*, we introduce a 2D histogram in the form of a heat map which shows the number of times each state occurs as an interim state in an infodesic; we here consider all ε-infodesics with ε<0.05 of the form $\psi_{s_{0}\to g}=\langle s_{0}=s,s_{1}=s^{\prime },s_{T}=g\rangle$ with non-repeating elements. The counts show that corner states, and to a lesser extent states along the edge or on the diagonal often participate as states in ε-infodesics. The agent is guided by optimal policies to move to the best vantage point with respect to information, a position where as many states as possible are informationally closer. This is consistent with the tendency of cognitive geometry, in our simple gridworlds with constant step penalties, to place corner and edge states more centrally for low-information-processing capacities (figure 3*b*,*c*).

We now proceed with restricting information processing to the nearly open-loop policy regime, *β* = 0.01 (figure 6). Note that one characteristic of such an open-loop scenario is to be able to reach all relevant goals with a single action distribution, which is the analogue of direction in our context.

Figure 6*a* shows the transition graph of the gridworld highlighting three states of the infodesic in green. The agent starts in the corner (*S* = #0) and is required to navigate to the adjacent state *g* = #1. The optimal policy to reach the adjacent final goal state is represented by the arrows in figure 6*b* with decision information as heat map and annotations. Since the transition function is not stochastic, the low tolerance for information cost, *β* = 0.01, means that the marginal action distribution has to generalize for as many states as possible. Hence, against naive expectation, significant probability is invested into actions moving away from the goal. The policy in state *S* = #0 retains a chance of moving out of the corner to the goal, although it is so small it is not visible on the plot, π(→|S=#0)≈0.016. The informational cost arising from this small difference in action distribution is clearly indicated in figure 6*b* where ${{ℑ}_{D}^{\pi_{F}}}_{1}(0)\approx 0.15$ bits, in contrast to the significantly lower decision information values in all other states.

The chance that the agent successfully leaves the corner in any given time step is very low as it predominantly chooses one of the prevailing actions in the policy (← or ↗), hits the edge, pays the cost of −1 in performance each time and remains in the corner without moving closer to the goal. This increased duration cost combined with the increased information cost results in a significantly higher free energy, $F_{1}^{\pi_{F}}(0)=73.9$, as shown in figure 6*c* which displays the same policy for the same goal (*S* = #1), but now annotated with free energy values.

It is both more performant and informationally more efficient to permit the agent to use an additional policy to facilitate leaving the initial corner state. To demonstrate this, we introduce an ε-infodesic where the interim state is diagonally adjacent to the initial corner (*S* = #8) as shown in figure 6*d*. The agent uses one policy for the intermediate subgoal (*s*_int_ = #8, policy displayed in figure 6*d*) and another policy from there to the final goal (*g* = #1, displayed in figure 6*c*). This cumulative free energy, when using two policies $F_{8}^{\pi_{F}^{\left(1\right)}}(0)+F_{1}^{\pi_{F}^{\left(2\right)}}(8)=10.92$, gives a drastic reduction of 85% in comparison to the free energy for using a single policy, leading to a quite substantial violation of the positivity of the triangle relation:

5.3 $$\frac{F_{1}^{\pi_{F}}(0)-(F_{8}^{\pi_{F}^{\left(1\right)}}(0)+F_{1}^{\pi_{F}^{\left(2\right)}}(8))}{F_{1}^{\pi_{F}}(0)}=-0.85.$$

The discussion will deal with the ramifications of introducing such switching policies.

## 6. Discussion

We first considered the concept of geometry in the sense of a collection of geodesics which derive from a quasi-metric and how they relate to the states of the given space and to each other. We then generalized this concept to the weaker notion of infodesics which take into account the cognitive costs. The discussion that follows develops the argument that the consideration of policy switching and that of infodesics are each valuable in their own right. Ignoring the cost of maintaining multiple policies, policy switching enables agents to plan policies on subsets of the state space instead of the entire state space at reduced information processing costs. Our take on infodesics is to consider them as a collection of sequences of states, e.g. landmarks which emerge even in systems that do not have obvious natural choke points (doors, etc.). We posit that this collection of infodesics can be used as a sparse representation of an environment.

In the traditional view of Euclidean geometry, one considers its structure which binds the various aspects together and enforces considerable constraints on its various components. In the case of straight lines, the geodesic in the Euclidean case can be defined by two distinct points in the space. Two such lines, if not parallel, define intersection points, and each of these intersections then defines an angle. Additionally, in Euclidean geometry one can ignore the directionality of straight lines due to their directional symmetry. When moving to Riemannian geometry, a few of these constraints (such as the sum of angles in a triangle) are relaxed or dropped entirely. In the case of a Finsler geometry, the underlying metric is further relaxed into a quasi-metric and the directionality of the geodesics becomes important owing to this asymmetry.

While these spaces can be derived by metrics or quasi-metrics, most of these spaces are not considered primarily in terms of distances, but in terms of a collection of the geodesics and how different geodesics relate to each other. However, here we adopted the perspective that, whatever structure one wishes to consider, it must derive from the costs of solving a task. In the case of deterministic value geodesics, this essentially reduces to deriving the latter from a quasi-metric.

Such considerations become especially important when moving to resource-rational approaches where cognitive costs matter. Being able to maintain a behaviour along a trajectory makes the task solution along a sequence of states cognitively cheaper, even if the pure distance cost may differ. When travelling to a goal, one has two components of cognitive operation. For the first, the policy, against which the given state needs to be monitored, secondly, some identificator trigger or cognitive feature that signals that the goal has been reached and the agent can stop.

Now, a point in favour of ‘desics’ is that there may be a suitable sensor or cognitive feature which will keep one on a ‘desic’ (e.g. keeping a compass direction, a fixed angle towards the sun or policy). By contrast, the ‘hitting’ of a goal state can be outsourced to another decision-making entity, e.g. a timer that estimates how much time remains before arriving at the goal, or some sensor triggered by the goal state, or, as in our case, by making the goal state (or subgoals, in the case of trajectory segments) explicitly absorbing. This means that following the desic itself is a low-information process as one is not required to keep track precisely of how far down a route one has moved. Stopping once a target is hit is considered a separate process, handled either by a separate cognitive or sensoric unit or by a trapping state of the environment itself. In other words, we separate this decision-making into a processual component and an identificatory component.

Before being able to capture that, we had first to adapt definitions to discrete spaces. So we proceeded to define geodesics by state sequences whose subsegments are distance-wise consistent with the overall sequence, i.e. which turn the triangle inequality into an equality. This works seamlessly for deterministic value geodesics. However, with the introduction of cognitive costs, when optimal policies can become probabilistic, the infodesic criterion based on the triangle inequality becoming an equality does not transfer smoothly. Instead, one obtains strongly impoverished infodesics, unless one relaxes the constraint. This, while violating the desirable properties associated with metrics or quasi-metrics, has some instructive implications which we discuss below.

Any structured strategy-finding reflects the intrinsic structure of the state space and its coherence rather than just carving out a single isolated trajectory from a specific starting state to a specific single goal. Understanding the structure of a task space thus inherently adopts a multi-goal perspective. To discuss its validity in predicting the decision-making behaviour of a resource-rational agent, consider, for instance, a person navigating to a destination while distracted or multi-tasking, perhaps they are talking on their phone while walking. In line with recent work on simplified mental abstractions [33] our expectation is that people will choose a less demanding route that will enable them to pay less attention to their exact location, and therefore plan a policy which can be more generally applied. Akin to compressing the state space, instead of storing a separate action distribution for each state, we can cluster the states and select a more universal policy which reduces the cost of selecting the appropriate actions in the different states. We can then ask which other goals can be achieved either equally optimally or with little extra effort with this particular action selection model, and extending this to the idea of agents switching goals on the fly while expending little additional computation.

When one generalizes from the problem of reaching a particular goal in a cost-only MDP to a general set of goals, one makes the transition from finding optimal trajectories with a specific end state to a whole arrangement of optimal sequences across the space. The idea remains the same. ‘Desics’ are in this perspective those sequences of states that are optimally reachable from the starting point. Geodesics are the most convenient of such collections as they have a metric or quasi-metric from which they can be derived. However, in this perspective, the ‘desic’ (geo- or infodesic) view does no longer require them to be actually derived from a strict metric or quasi-metric, although for better interpretability we still strive to derive it from a concept that is as close to the quasi-metric as possible.

### 6.1. Generalizing geodesics

When thinking in terms of geodesics one can, instead of considering it an optimal route between two states in the space, select these by choosing a starting state *s* and a generalized ‘direction’. We reiterate that in our framework we generalize the choice of a fixed compass direction to the choice of an open-loop policy. Following that policy fixedly will keep us moving in a particular direction towards the goal. This is best illustrated by way of an example: when humans navigate they often abstract the route; for example, assume that the shortest path runs along a fixed compass direction, say, North. If they later find they need to also visit a waypoint which happens to be between the starting point and the goal, they profit from the fact that their selected policy is also optimal for any waypoints in between. This means that all states on that geodesic, and not just *g*, are reached optimally from *s* under this policy. Identifying geodesics determines an optimal trajectory of goal states along the way and thus solves a whole class of problems at once, which is a critical motivation for the geometric perspective.

We point out a dichotomy between considering ‘shortest paths’-type and ‘constant directions’-type criteria. The shortest path route is attained by the optimization of the value term in the free energy, while the optimization of the decision information term corresponds to minimizing the state-specific deviation of the action distribution from the overall marginal. The latter can be seen as an attempt to make the policy approach an open-loop policy as closely as possible. In this interpretation, cognitively cheap routes correspond to the direction-preserving trajectories. This means that one can interpret a free energy optimization at given *β* as selecting a trade-off between shortest-route and direction-preserving ‘desics’.

We can obtain geodesics based on shortest routes from the optimal trajectories between a state *s* and a goal *g* whenever they are derived from a value function and executed as deterministic trajectories. On the one hand, the Bellman property guarantees that for any intermediate state *s*′ on such a trajectory the *remaining* part of the trajectory from *s*′ to *g* must be optimal; for a proper interpretation as a geodesic, we also require the counterpart: the *preceding* portion leading from *s* to *s*′ should be optimal.

We propose that this perspective throws a different light on the organization of tasks, namely, instead of considering the task solution simply as a ‘recipe’ that is carried out starting in a state to reach a goal, with the Bellman equation merely providing a computational advantage, it rather defines a similarity and an overlap of the ‘basin of attraction’ of nearby goals. If one considers its dual, namely the ‘zone of repulsion’ around the starting state, this leads naturally to the concept through which we define the geodesics: namely that the *initial* segment (*s* to *s*′) of a geodesic must also be optimal. This is the motivation for our definition of the generalized geodesic as one where the (generalized) triangle inequality (4.1) must hold as an equality for a sequence of the elements of the geodesic.

In analogy to the basin of attraction which creates concepts of similarity for goals via the Bellman property, the zone of repulsion thus creates such a similarity for starting states via the requirement of optimality for the initial segment. In other words, one obtains a natural structure of task similarities and a set of relations with respect to each other, rather than treating the task of reaching a given goal as a completely different undertaking for each distinct goal. Additionally, the concept of geodesics creates a class of goals which are solved by maintaining the same policy. This means that, even if the goals are not close to each other, they belong in the same class of problems being solved by the same policy.

This criterion renders the totality of the elements of the sequence following this policy optimally reachable from the starting point. With added information processing constraints in the form of decision information, this generalizes the existing concept of pure value geodesics to *infodesics*. By using this term we imply the totality of the infodesics, their relation to the various states in the state space, and with respect to each other. This creates characteristic distortions in the geometry of the problem under consideration and defines what we call our *cognitive geometry*.

A cognitive geometry introduces some novel effects. With reduced information processing capacity, optimal policies typically become stochastic, which can render infodesics trivial, i.e. only *s* and *g* themselves fulfil the triangle equality criterion. We, therefore, included relaxed versions of the equality to still be able to consider near-infodesics. However, further drastic deviations are possible, such as in (5.3), where splitting a trajectory into two parts governed by different policies can significantly *reduce* the total cost. This corresponds to switching policies at the intermediate state *s*′. The latter means that one uses two different marginal action distributions throughout the run and thus enables the execution of a more complex overall policy than with a single trajectory. Notably, and as a new phenomenon, this reduction in information only appears because the original triangle inequality does not include any complexity cost involved in a policy switchover in decision information, despite the fact that this requires actual cognitive effort. We thus expect that a complete characterization of cognitive geometry will need an expanded form of the triangle inequality that will take such policy switching costs into account in a systematic way.

### 6.2. Free energy as a quasi-metric?

Ideally one would have liked the infodesics to derive from a quasi-metric. When we consider free energy against the requirements for a quasi-metric space, we find the following:

D1 Non-negativity: we have already shown that $F_{g}^{\pi_{F}}(s)\geq 0$ as $V^{\pi_{F}}(s)\leq 0$ and ${{ℑ}_{D}}^{\pi_{F}}(s)\geq 0$, where $\pi_{F}$ is optimized in terms of free energy for goal state *g*.
D2 Principle of indiscernibles: we know that $\forall g\in GF_{g}^{\pi }(g)=0$ for all *π* as goal states are absorbing and for $s\in S\setminus g,F_{g}^{\pi }(s)\neq 0$ for all *π*.
D3 Asymmetry: in general, the optimal policy navigating from $s\overset{\pi_{F}^{\left(1\right)}}{⟶}g$ differs from the policy navigating from $g\overset{\pi_{F}^{\left(2\right)}}{⟶}s$. Therefore, ${{ℑ}_{D}}^{\pi_{F}^{\left(1\right)}}(s)\neq {{ℑ}_{D}}^{\pi_{F}^{\left(2\right)}}(g)$ and hence $F_{g}^{\pi_{F}^{\left(1\right)}}(s)\neq F_{s}^{\pi_{F}^{\left(2\right)}}(g)$ in general, making free energy at best a quasi-metric.
D4 Triangle inequality: we ask whether $F_{g}^{\pi }(s)\overset{?}{\leq }F_{s^{\prime }}^{\pi_{F}^{\left(1\right)}}(s)+F_{g}^{\pi_{F}^{\left(2\right)}}(s^{\prime })\;\forall s\in S$, where each segment uses a different optimal policy $\pi_{F}^{\left(1\right)}$ and $\pi_{F}^{\left(2\right)}$.

For the free energy to form a quasi-metric, the crucial axiom to establish is that the triangle inequality holds. However, we have already demonstrated with a counterexample that the partitioned cumulative free energy can be lower than the free energy without subgoaling (5.3). Hence, in general, the triangle inequality does not hold and the free energy is not a quasi-metric.

### 6.3. Task decomposition

*Rational analysis* involves understanding human cognition as a rational adaptation to environmental structure and it is extended by *resource-rational analysis* which aims to explain why people may choose to adopt particular goals or heuristics in view of their cognitive resources [8]. People’s cognitive strategies are jointly shaped by the environment and computational constraints when planning strategies to complete a task [34]. Maisto *et al.* [35] propose that, during planning, intermediate goals are selected to minimize the computational Kolmogorov complexity. Resource-rational task decomposition, which reduces the computational overhead of planning at a primitive action level, is consistent with elements of previous studies on human planning [36]. Ho *et al.* [33] conducted trials of candidates navigating a simple gridworld through a variety of obstacles to reach a goal which demonstrates that people plan by constructing a simplified mental representation of the task environment [33]. Here, we propose a complementary interpretation, where making use of segmentation by incorporating interim goals one reduces informational costs of the individual infodesic routes.

In our context, consider the classical mountaineering techniques known as ‘aiming off’ and ‘handrailing’ [37], which are often combined in poor visibility. Aiming off entails subgoaling in the form of deliberately aiming away from the intended goal instead of trying to walk on an exact bearing, for example, aiming due East of the goal. Handrailing is when the mountain leader walks along a feature of the landscape, i.e. a cliff, fence, etc. This provides robust and cognitively cheap navigation. For example, an interim goal is selected which is known to be due East along a fence from the actual intended destination. The mountain leader aims off to the interim goal which is known to be due East of the final goal, arriving at this interim goal, one then knows the final goal is due West along the fence. The mountain leader then handrails the fence to arrive at the final destination. This task decomposition enables a person to benefit from a reduction in the information processing required due to the structure of the environment, i.e. an epistemic strategy: physical actions ‘that [make] mental computation easier, faster or more reliable’ [38].

When segmenting an infodesic, one decomposes the original problem into subproblems with given subgoals. The constituent free energies for these subgoals use different policies. In the deterministic case with greedy value optimization (*β* → ∞), no state will be revisited twice throughout a trajectory. This means that we can always construct a superpolicy for the overall goal-seeking behaviour by integrating the respective subpolicies and policy switching is not required. Even if we consider lower *β* values, whenever there is no ambiguity about which subpolicy applies at which state, i.e. no overlapping of the support for the different policies, the validity of the triangle inequality for free energy can be upheld, as shown in the theorem presented in the electronic supplementary material, S9. However, when we reduce *β*, policies typically become increasingly stochastic to reduce the divergence from the action marginal. At some point, the stochastic trajectories belonging to different subgoals will begin to overlap en route: the agent may visit the same state during different segments of the infodesic, and therefore under different policies. At the given state it is thus now no longer uniquely resolvable without further information as to which of the subsegment policies is currently active.

As we have shown, although without taking any switching costs into account, this enables the agent to benefit from informationally more efficient policies, having an advantage over single policies by better making use of the structure of the environment (similar to temporal abstractions in [39] and subgoaling in [40]). For instance, as can be seen in the example shown in figure 5*b*, by moving to the corner opposite the goal, the agent aligns all future actions with the marginalized action distribution thereby reducing the cost of information processing even while incorporating the sequence 〈#0, #6, #12, #18〉. To ascertain which is cognitively cheaper will require a more thorough understanding of the switching costs. It is possible that there are cases when it may be cheaper to use multiple policies even when taking the switching costs into account.

### 6.4. Grid cells and cognitive maps

It is becoming increasingly central to study the geometry of perception and decision-making [41,42]; thus, one reason to strive towards a formalization of cognitive geometries is the long-standing idea that humans and animals form cognitive maps of their environment [43]. A large body of neurophysiological work has demonstrated that the hippocampus is sensitive to the geometry of the environment, suggesting that it may support the formation of cognitive maps [44]. Two cell types are of particular interest in this: place cells which fire at unique locations in the environment [45] and grid cells which fire at multiple locations forming a regular, triangular lattice [46]. The former is suggested to enable topological navigation while the latter may provide a spatial metric supporting path integration and vector-based navigation [47].

While there is strong evidence to suggest that the hippocampus is involved in spatial navigation, growing evidence suggests that the representation of location in itself may not be the sole purpose and that the hippocampal formation may instead also support other important functions, such as reward-guided learning [48,49]. Since success in RL does not rely on Euclidean distance, but the distance along paths (e.g. around obstacles), efficient RL should thus rely on a geodesic rather than a plain Euclidean metric [50]. In a series of simulations, Gustafson & Daw [50] provide theoretical support for the benefit and empirical evidence of this spatial coding and hypothesize that the firing of place and grid cells may be modulated accordingly.

In line with this work, we here additionally speculate that it is not only a purely geodesic metric that is of importance to biological agents, but that additional cognitive costs, which we model by information-theoretical quantities, should also affect computations and hence be supported by the underlying metric. Since the hippocampus is also involved in the representation of information like value [51] and goals [48,49,52], or even conceptual spaces [53,54], it appears that activity of cells found in the hippocampal formation may reflect geometrical properties more generally. In this light, it appears not without reason to hypothesize that this brain area may also be a good candidate to probe for its sensitivity to the geometry that arises from a trade-off between value and information as demonstrated in this work.

### 6.5. Future work

For a complete model of the concatenation of sub-infodesics, it will be necessary to model the detection of a subgoal being achieved, together with a memory state of the agent that switches to keep track of the currently active subgoal. We expect an informational overhead involved with the policy switching costs *C*_switching_ for this infodesic composition needs to be incorporated in the overall costs. We conjecture *C*_switching_ to be most aptly modelled as a cost that captures the information indicating which subgoal is currently active, or, more precisely, the cumulated cost of checking the currently active policy, for each decision taken, since we have a memoryless agent. We posit that, while the total decision complexity is reduced by switching between multiple policies, when a suitably defined cost *C*_switching_ of maintaining these multiple policies is included, we will obtain a ‘relaxed’ triangle inequality of the form: $F_{g}^{\pi_{F}}(s)<=F_{s^{\prime }}^{\pi_{F}^{\left(1\right)}}(s)+F_{g}^{\pi_{F}^{\left(2\right)}}(s^{\prime })+C_{\text{switching}}$, which takes this switching cost into account and would be the necessary condition for using the free energy as a relaxed quasi-metric. This switching cost might be characterizable systematically from the properties of the task space. If verified, it would be an interesting target for investigation to study how switching costs could contribute to identify particularly cognitively convenient states in the task space.

Information-to-go is only one very specific measure and it might be insightful to study other resource-rational measures in a geometric context instead. This will possibly require other adaptations of the triangle inequality. These could provide ways of comparing possible preferred behaviour segmentations under these measures with experimental observations.

Our scientific objective is to use this framework as a model for the way in which bounded rational agents, human and robots, map their knowledge of interrelations between states. In the context of navigation this would be how to travel efficiently between known locations using previously planned optimal routes. An additional experiment that would allow one to confirm the cognitive plausibility of this framework is inspired by the trials conducted by Ho *et al.* [33]. The experiment involves a navigation task in a virtual maze solved by human subjects under information processing constraints, which are induced by requiring that candidates recall their routes at the end of the task. Candidates will be surveyed regarding their estimation of the distances between pairs of states in the environment. Our hypothesis is that once the experiment has been conducted, the distances estimated by the candidates will correspond to the distances predicted by the proposed cognitive geometry.

When humans plan a route, they often do so with previously experienced and memorized routes in mind and using these as points of reference to explore unknown territory. Then, upon implementing the plan, they assess the route and depending on their evaluation they either store the information to be used again or discard the route. In other words, we have the implicit assumption that the ‘desics’, rather than some random routes, serve as a natural skeleton from which to look for ways to explore the unknown parts of the space.

## 7. Conclusion

We considered distances induced by cost-only MDPs which were additionally endowed with an informational cost reflecting the complexity of decision-making. Geodesics generalize the intuition about geometry determined by directions and distances, representing optimal transitions between states. We proposed that the addition of informational criteria would characterize a *cognitive geometry* which additionally captures the difficulty of pursuing a particular trajectory.

We found that free energy retains some of the structure of the spatial geometry via the value function while incorporating the cost of information processing. However, emphasizing the most informationally efficient policy among otherwise equivalent policies will favour trajectories which pass through informationally efficient states en route to the goal. Such trajectories, therefore, often experience a ‘detour’ in terms of pure distance through more easily manoeuvrable hubs. In specific examples we found considerable distortions which place boundary states more centrally in the space, with the boundaries acting as guides. Additionally, when considering infodesics, i.e. sets of intermediate states which are optimally reachable from the starting state, intermediate goals can be achieved en route to the final goal, thus defining classes of problems that are solved as a side effect of solving the main one.

Analogously to geodesics, we characterized the infodesic property by the triangle inequality becoming an equality. Since this inequality is not always perfectly respected in our framework, we had to relax the conditions. Furthermore, due to the informational nature of the free energy distance, splitting a trajectory devolves the possible cost of switching the policies of the two segments into the split itself. This can cause the violation of the triangle inequality to be quite significant. Note that one can separate the contributions of the trajectory cost and the splitting cost and thus to understand (and control) how much each of these contributes to trace how much of the complexity is found in the trajectory and how much in the splitting. In the future, the splitting cost will be explicitly incorporated into a generalized, but tighter triangle inequality for a more stringent description of the infodesic.

Establishing such a cost, we propose, will allow us to impose, with its quasi-distance structure and additionally the ‘directional’ structure induced by the policy choice, a quasi-geometrical signature on the state space. We suggest that this offers the basis of a genuinely geometrical notion of task spaces that takes into account cognitive processing: a cognitive geometry. This we understand to be a structure with optimal trajectories determined either by two states or by one state and a ‘direction’ (i.e. policy) that is informed not only by the pure spatial geometry, but also by the cognitive costs that an agent needs to process when moving from task to task and how it has to informationally organize policies to achieve nearby or related tasks. Critically, such a concept would suggest that straightforward use of Euclidean, or geodesic-based geometry may not be the appropriate language to treat even purely navigational decision problems. For that reason, it would be interesting to investigate this distance further.

## Data Availability

The data, source code and sample Python notebooks related to this work are publicly available from the research group repository at https://gitlab.com/uh-adapsys/cognitive-geometry and also archived within Zenodo: https://doi.org/10.5281/zenodo.7273868 [55]. The data are provided in electronic supplementary material [56].

## Footnotes

1 By assuming that the full task trajectory from *s* to *g* is a cheapest or shortest route, we ignore without loss of generality any technical complications from a possible wraparound of the trajectory.
